# Book Reviews

**Published:** 1914-03

**Authors:** 


### BOOK REVIEWS

ANATOMY AND PHYSIOLOGY FOR NURSES.—By LeRoy Lewis, M. D., formerly Surgeon to and Lecturer on Anatomy and Physiology for Nurses at the Lewis Hospital, Bay City, Mich. Third Edition, revised thoroughly. 12mo of 326 pages, with 161 illustrations. Philadelphia and London. W. B. Saunders Company, 1913. Cloth $1.75 net.

The cordial reception of the first edition of this book led its author to adhere closely to the original plan and manner of presenting the subject.

---

A CLINICAL MANUAL OF MENTAL DISEASES.—By Francis X. Dercum, M. D., Ph.D. Professor of Nervous and Mental Diseases, Jefferson Medical College, Philadelphia. Octavo of 425 pages. Philadelphia and London. W. B. Saunders Company, 1913. Cloth $3.00 net.

Dercum has prepared this book from the annual course of lectures delivered at the Jefferson College in a very practical manner. Emphasis has been placed on the clinical pictures, prognosis and treatment.

